# A Single-Layer Full-Color Diffractive Waveguide by Lithography

**DOI:** 10.3390/nano16010006

**Published:** 2025-12-19

**Authors:** Yong Li, Fei Wu, Huihui Li, Haitao Yang, Mengguang Wang, Zhenrong Zheng

**Affiliations:** 1 College of Optical Science and Engineering, Zhejiang University, Hangzhou 310027, China; liyong@llvision.com (Y.L.); wufei@llvision.com (F.W.); wangmengguang@zju.edu.cn (M.W.); 2 Beijing LLVision Technology Co., Ltd., Beijing 100176, China; lihh@llvision.com (H.L.); yanght@llvision.com (H.Y.)

**Keywords:** hyperspectral imaging, compressive sensing, filter array, single-layer diffractive waveguide

## Abstract

Augmented reality (AR) near-eye displays (NEDs) couple microdisplay image light to the human eye via integrated optical modules, enabling seamless virtual–real fusion. As core components that synergistically transmit and diffract light, diffractive waveguides are promising for next-generation AR NEDs but face two bottlenecks: compromised full-color performance in single-layer structures caused by grating dispersion and lack of scalable fabrication technologies. To address these, we first propose a mass-production-compatible workflow based on deep ultraviolet (DUV) lithography for large-area nanostructured optics. This workflow enables high-precision wafer-level production with 200 mm wafers and nine dies per wafer, overcomes scalability issues, and is fully compatible with straight-configuration nanostructures to ensure manufacturing feasibility. Leveraging this workflow, we develop a single-layer diffractive waveguide system for AR NEDs, which comprises a thin glass substrate, a broadband high-efficiency multi-layer dielectric in-coupler, and a 2D out-coupler that concurrently expands and out-couples light. Rigorous coupled wave analysis (RCWA) optimized coupler diffraction, while ray tracing refined guided light intensity and significantly improved exit pupil uniformity. This work establishes a foundation for full-color, high-efficiency AR waveguides and provides a scalable paradigm for large-area nanostructured optical systems such as telescopes and lithography equipment.

## 1. Introduction

Augmented reality (AR) near-eye displays (NEDs) couple microdisplay images to the human eye via optical components, enabling seamless virtual–real fusion and rapid expansion from military to consumer applications such as AR glasses and headsets [1]. Various optical solutions have been developed for NEDs, including free-form prism optics [2], beam splitter-based bird-bath optics [3], geometric waveguides [4,5], volume holographic grating waveguides [6,7], and surface-relief grating waveguides [8,9,10,11,12,13]. However, these technologies face inherent limitations: free-form prism and bird-bath optics attenuate ambient light, reducing real-world scene transmittance; geometric waveguides demand ultra-high alignment accuracy for reflective mirror arrays, hindering mass production; and volume holographic grating waveguides suffer from poor uniformity, fabrication challenges, and a limited field of view, such as Digilens’ 50° maximum field of view (FOV) due to constrained refractive index modulation [7,14].

Diffractive waveguides hold great promise for next-generation AR NEDs due to their synergistic light transmission and diffraction capabilities, yet they are hindered by two critical bottlenecks [15,16]. First, asymmetric in-couplers such as slant and blazed gratings exhibit strong angular and wavelength selectivity despite their high diffraction efficiency [17,18]. More importantly, the absence of cost-effective and scalable fabrication techniques constrains the manufacturing of large-area nano-optical structures [19,20]. Prior fabrication attempts, including nanoimprint lithography (NIL) and stepper lithography, have failed to mitigate these challenges [21]. NIL suffers from pattern defects, low throughput, and template wear, while stepper lithography is limited to small apertures that are incompatible with complex diffractive patterns [22].

**Figure 1:**
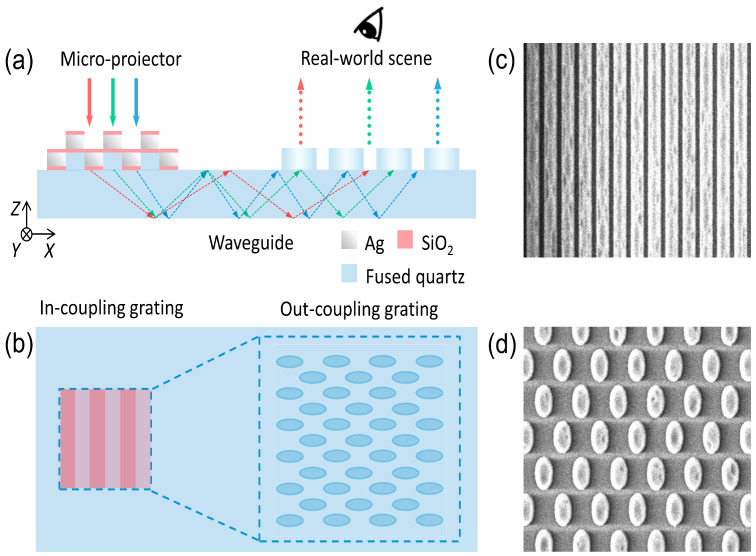
Schematic of the diffractive waveguide. (**a**) Side view illustrating light coupling from the micro-projector, TIR propagation in the fused quartz substrate, and out-coupling to the eye, alongside unobstructed transmission of real-world light. The structure integrates Ag, SiO_2_, and fused quartz components. (**b**) Top view of the strip-shaped in-coupling grating and specially designed two-dimensional out-coupling grating. (**c**,**d**) Scanning electron microscopy (SEM) images of the in-coupler and out-coupler nanostructures, respectively.

To tackle these challenges, this work presents a mass-production-compatible micro/nano patterning process based on 248 nm projection lithography—tailored for large-area metasurface grating waveguides [23]. This technology overcomes traditional fabrication limitations by eliminating template wear and defects, ensuring high throughput, and enabling the high-precision patterning of large-area substrates while supporting diverse micro/nano-structural designs [22,24]. Leveraging this workflow, we developed a single-layer full-color diffractive waveguide system for two-dimensional beam expansion, integrating a thin flat glass substrate, a broadband high-efficiency multi-layer dielectric in-coupler, and a two-dimensional out-coupler that concurrently expands and couples out light [25,26], as shown in Figure 1.

Guided by the *k*-vector condition, each grating’s rotation angle and period were precisely tailored to achieve distortion-free beam expansion, in-coupling, and out-coupling while preserving wavefront integrity—addressing the non-linear dependence of grating diffraction angle on incident angle. As shown in Figure 1a, the waveguide integrates a broadband high-efficiency in-coupler with a four-layer dielectric structure: 80 nm of high-refractive-index glass, 20 nm of silicon dioxide (SiO_2_), 100 nm of silver, and 20 nm of SiO_2_. Optimized via simulation for red, green, and blue (RGB) efficiency and angular uniformity, this design overcomes the angle and wavelength selectivity of traditional blazed and tilted gratings and enables mass production through 248 nanometer projection lithography’s precision. Figure 1b illustrates the out-coupler’s elliptical region array design for efficient light extraction and uniformity, with SEM images in Figure 1c,d validating both couplers’ structural fidelity—well-defined in-coupler strips and uniform out-coupling units—and compatibility with 248 nm projection lithography. Detailed specifications of the in-coupler’s layer thicknesses and the deposition methods for the multi-layer structure are provided in Section 3 and Section 4, respectively. This integration of innovative fabrication and structural optimization resolves the mass-production bottleneck for large-area AR waveguides, enhances optical performance, and advances the commercialization of consumer-grade AR NEDs.

## 2. Theoretical Fundamentals of Diffractive Waveguide

To establish the design basis for the diffractive waveguide, we first define the theoretical framework governing light-grating interactions and FOV constraints. The direction of input collimated light to the gratings can be described by the polar angle θ and azimuthal angle ϕ. The input wave vector $\vec{k}$ can be defined as

(1) $$\vec{k}=\frac{2\pi }{\lambda_{0}}\left(nsin\theta cos\phi \hat{x}+nsin\theta sin\phi \hat{y}+ncos\theta \hat{z}\right)$$

where $\lambda_{0}$ denotes the vacuum wavelength, *n* is the substrate refractive index, and $\hat{x}$, $\hat{y}$, and $\hat{z}$ are the unit vectors along the *x*, *y*, and *z* axes, respectively. The grating direction vector $\vec{G}$ is similarly described using its polar angle $\theta_{G}$, azimuthal angle $\phi_{G}$, and period Λ:

(2) $$\vec{G}=\frac{2\pi }{\Lambda }\left(sin\theta_{G}cos\phi_{G}\hat{x}+sin\theta_{G}sin\phi_{G}\hat{y}+cos\theta_{G}\hat{z}\right)$$

The diffraction distribution of the *m*^th^-order diffracted light is quantified by the relative direction cosines, with superscripts transmission *T* and reflection R distinguishing propagation modes:

(3) $$\alpha_{m}^{T,R}=nsin\theta cos\phi +\frac{m\lambda_{0}}{\Lambda }sin\theta_{G}cos\phi_{G}$$

(4) $$\beta_{m}^{T,R}=nsin\theta sin\phi +\frac{m\lambda_{0}}{\Lambda }sin\theta_{G}sin\phi_{G}$$

(5) $$\gamma_{m}^{T}=ncos\theta +\frac{m\lambda_{0}}{\Lambda }cos\theta_{G}$$

(6) $$\gamma_{m}^{R}=-ncos\theta +\frac{m\lambda_{0}}{\Lambda }cos\theta_{G}$$

Equations (5) and (6) define the third direction cosine γ for the mth-order diffracted light, where superscripts T and R correspond to transmission and reflection grating cases, respectively. For the transmission case (γ_m_^T^), the direction of the diffracted light along the *z*-axis is consistent with the incident light; for the reflection case (γ_m_^R^), the *z*-axis direction of the diffracted light is opposite to the incident light. This assumption is based on the basic propagation characteristics of transmission and reflection gratings in diffractive waveguides. Notably, the FOV of optical engines and NEDs is typically specified in Cartesian coordinates (α, β), where *α* and *β* represent angles relative to the *x* and *y* axes, respectively. To calculate diffraction distribution, incident light coordinates (α, β) must be converted to spherical coordinates (θ, ϕ) using

(7) $$\tan\alpha =\frac{sin\theta cos\phi }{cos\theta }$$

(8) $$\tan\beta =\frac{sin\theta sin\phi }{cos\theta }$$

**Figure 2:**
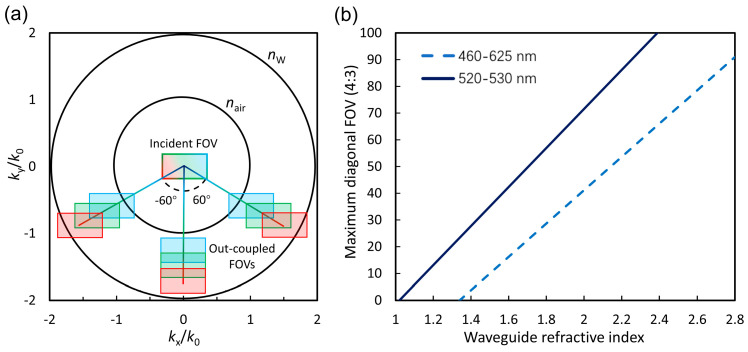
*k*-vector diagram of FOV and spectral diffraction efficiency. (**a**) *k*-vector diagram defining the diffractive waveguide’s valid FOV, bounded by air and substrate refractive indices, confirming a 40° FOV for full-color operation with n_w_ = 2.0; (**b**) shows the diffraction efficiency distribution across RGB wavelengths, verifying broadband compatibility. Both subparts validate the waveguide’s FOV control and spectral performance for AR NEDs.

Grating diffraction angles depend non-linearly on incident angles, so each grating’s rotation angle and period must be precisely designed per the *k*-vector condition to achieve distortion-free beam expansion, in-coupling, out-coupling, and wavefront integrity—this logic is visualized in Figure 2a [10]. Guided by the *k*-vector principle, the waveguide’s valid FOV is bounded by the total internal reflection (TIR) region, defined by air’s refractive index *n*_air_ as the inner radius and the substrate’s refractive index *n*_w_ as the outer radius. A substrate with a high refractive index of 2.0 is adopted to transmit full-color red, green, and blue images through the single-layer waveguide, as it expands the TIR region to support a 40° FOV. Figure 2b clearly illustrates the scope of this region and the 40° FOV range, confirming the substrate’s suitability for AR NEDs. For efficient two-dimensional beam expansion and image out-coupling to the human eye, the out-coupler uses a hexagonal distribution and a specialized two-dimensional diamond-shaped grating structure—formed by superposing two one-dimensional gratings oriented at 60 degrees, resulting in a horizontal to vertical period ratio of √3:1. The sum of the grating vectors is set to zero to ensure consistency between coupled-in and coupled-out beams and avoid image distortion.

## 3. Waveguide Design and Simulation

**Figure 3:**
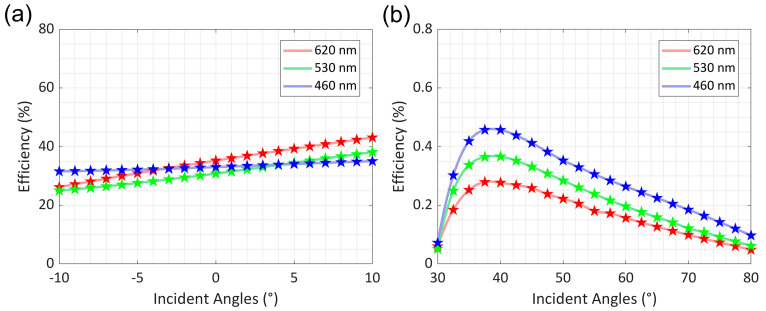
The coupler diffraction efficiency of the in-coupler and out-coupler at different angles of RGB. (**a**) In-coupler efficiency form −10° to 10° incident angles for blue (460 nm), green (530 nm), and red (620 nm). (**b**) Out-coupler efficiency from 30° to 80° for the same wavelengths. The curves are interpolations based on RCWA, and the dots are raw data.

Simulation results in Figure 3 further validate the couplers’ optical performance. For the in-coupler results in Figure 3a, tested over the −10° to 10° incident angle range matching typical AR optical engine inputs, the diffraction efficiency averages are 35% for red 620 nm and blue 460 nm wavelengths and 31% for green 530 nm. More importantly, RGB efficiency variation stays below 8% across the angle range, directly resolving traditional single-layer waveguide full-color distortion and eliminating the need for multi-piece gratings. For the out-coupler, characterized over the 30° to 80° range matching substrate TIR propagation angles, diffraction efficiency remains stable for all three primary colors with no significant drops at the FOV edges. Consistency between RCWA-based interpolation curves and raw data confirms simulation reliability, while efficient uniform performance across angles and wavelengths verifies that the couplers meet immersive AR near-eye display core optical requirements.

When total reflection light hits the coupling grating, most transmits as zero-order R(0, 0), continuing incident propagation. The two-dimensional grating also diffracts light into first-order light, bent ± 60° relative to the *x*-axis to interact with other diamond-shaped gratings as R (1, 1) and R (1, −1). Second-order diffractive light from the two-dimensional hexagonal grating couples out the waveguide toward the observer as T (0, 2), a distribution exactly confirmed by RCWA. The diamond-shaped grating has a 720 nm major axis and 416 nm minor axis, with stable RGB diffraction efficiency over the full 30° to 80° FOV incident angle range, as shown in Figure 3b. The elliptic two-dimensional grating design enables bidirectional beam expansion and enhances pupil brightness uniformity.

## 4. Fabrication and Measurement Experiments

**Figure 4:**
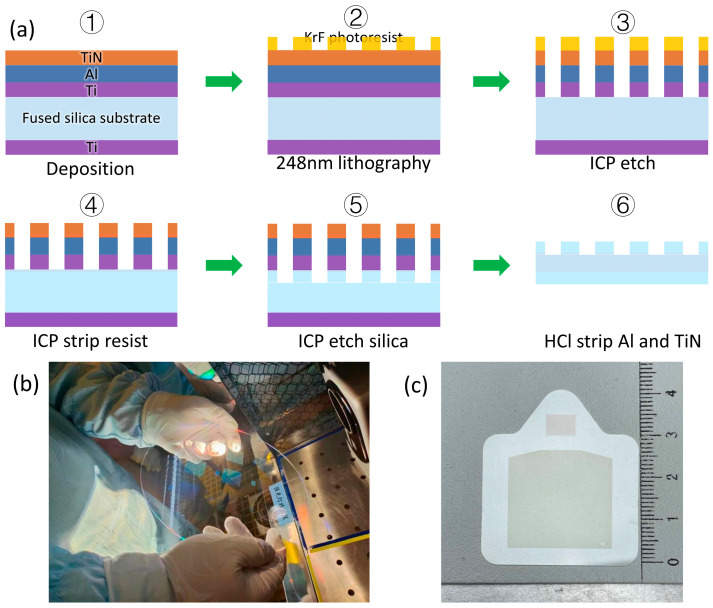
Diffractive waveguide fabrication workflow. (**a**) This figure illustrates the 8-inch wafer-scale DUV lithography process: multi-layer deposition on fused silica, 248 nm lithography, ICP etching, and residual metal cleaning. (**b**) This image shows an 8-inch quartz wafer integrating nine waveguide units for wafer-level mass production, ensuring consistent optical performance for consumer-grade AR devices. (**c**) A physical picture of a single optical waveguide, with the ruler shown in the image marked in centimeters.

To meet the optical requirements of AR NEDs, the diffractive waveguide is functionally divided into two optimized regions: a 7 mm × 5 mm in-coupler, sized to match the optical engine’s pupil diameter for efficient light collection, and a 24.6 mm × 23.4 mm out-coupler engineered to cover the eye box at the designated eye relief. For scalable manufacturing, nine such waveguide units are integrated onto an 8-inch quartz wafer, as visualized in Figure 4b, enabling wafer-level mass production critical for consumer-grade AR device commercialization.

The waveguide and its meta-grating components were fabricated via a customized 248 nm deep ultraviolet lithography (DUV) workflow using a Nikon NSR S205C stepper (Tokyo, Japan), with the complete process detailed in Figure 4a. Leveraging the high precision and yield of integrated-circuit (IC)-compatible lithography, the workflow follows a sequential process: (1) substrate preparation using a 725 μm thick quartz wafer; (2) multi-layer film deposition, including 100 nm titanium (Ti) sputtered on the back side to convert transmission to reflection for process compatibility, and a front-side stack comprising a 10 nm Ti adhesion layer, 150 nm aluminum (Al) reflection layer, and 20 nm Ti nitride anti-reflection coating; (3) photolithography, involving spin-coating of 400 nm UV-135 KrF positive photoresist, pre-baking at 120 °C for 60 s, deep ultraviolet exposure, post-exposure baking at 130 °C for 60 s, and development in a 0.26 N CD-26 developer for 45 s; (4) inductively coupled plasma (ICP) etching with tailored gas flows and power parameters for TiN, Ti, and quartz; (5) residual metal removal via cleaning with a mixture of 29% ammonia water, 30% hydrogen peroxide, and deionized water in a 1:1:4 volume ratio; and (6) final lift-off and removal of Ti and Al for meta-grating patterning, implemented through plasma-enhanced chemical vapor deposition (PECVD), photoresist application, electron beam lithography (EBL) patterning, and chromium physical vapor deposition (PVD) as preceding steps. This complete sequential process ensures the structural fidelity of the meta-gratings and enables reliable experimental characterization of their diffraction efficiency, beam expansion capability, and integration compatibility with the waveguide, laying a solid foundation for validating the overall optical performance of the AR near-eye display component.

**Figure 5:**
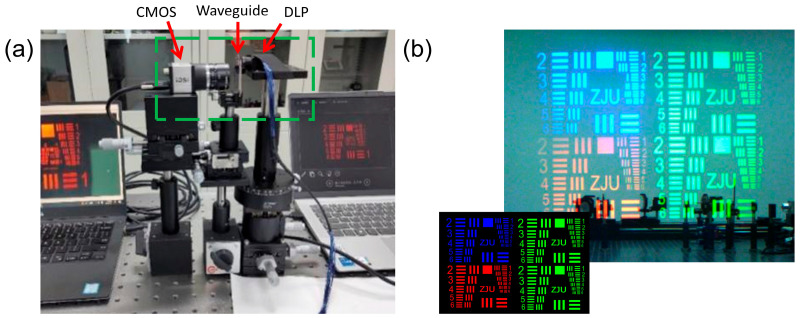
Experimental setup and AR image results. (**a**) Key components (DLP, waveguide, CMOS) are labeled in the physical experimental setup here. The setup includes a DLP optical engine (1280 × 720p resolution, 40° FOV), a diffractive waveguide positioned between the DLP engine and the optical path, an 18MP CMOS camera, and an 8mm Tamron lens. The Tamron lens is the black barrel-shaped component placed in front of the CMOS camera. Light emitted by the DLP optical engine couples into the waveguide, and outgoing light from the waveguide is imaged by the Tamron lens onto the CMOS camera for signal acquisition. (**b**) AR images captured by CMOS, showing clear RGB patterns such as “ZJU” with retained details, verifying the waveguide’s full-color display capability.

Optical performance validation was conducted using a custom experimental system illustrated in Figure 5a. The system comprises a CoreTronics digital light processing (DLP) (Hsinchu City, Taiwan) optical engine with 1280 × 720p resolution, 40° diagonal FOV, 200:1 contrast and RGB LED light source, the fabricated diffractive waveguide, an IDS UI-3590CP 18MP CMOS camera (Obersulm City, Germany) with a 1.25 μm pixel size, and an 8 mm Tamron lens (Saitama City, Japan). The captured AR images shown in Figure 5b demonstrate clear full-color RGB pattern reproduction with retained details, confirming the waveguide’s full-color display capability. The actual eye box size of this waveguide system, validated via both simulations and experiments, is determined to be 12 mm × 12 mm, which meets the practical viewing requirements of consumer-grade AR devices. Under the 40° diagonal FOV (16:9 aspect ratio), the system achieves a maximum optical efficiency of 246 nits/lm, exhibiting favorable brightness output capability. During image capture, the effective aperture of the IDS UI-3590CP CMOS camera was adjusted to 3–4 mm, which matches the pupil diameter of the human eye under typical daily lighting conditions.

## 5. Conclusions

This paper develops a lithography-based manufacturing process suitable for large-scale production and, based on this process, proposes a diffractive waveguide system for two-dimensional beam expansion—an essential component for AR lenses. This process is scalable: it allows for the fabrication of diffractive waveguides on 8-inch wafers, each integrating nine waveguide units. The straight nanostructures of the in-coupler and out-coupler are well suited for photolithography, which ensures manufacturing feasibility and efficiency. The waveguide system itself integrates a single thin flat glass substrate with a broadband high-efficiency multi-layer dielectric in-coupler and a two-dimensional out-coupler that concurrently achieves beam expansion and light out-coupling. The fabricated waveguides retain a precise outline and grating period, which guarantees high-resolution AR image display. Moreover, the developed lithography process supports the highly scalable, cost-effective mass production of large-aperture metalenses and can be extended to other large-scale nanostructured patterns, ultimately paving the way for the development of full-color, high-efficiency diffractive waveguide systems for AR applications.

## Data Availability

The original contributions presented in this study are included in the article. Further inquiries can be directed to the corresponding author.

## Abbreviations

The following abbreviations are used in this manuscript:

AR	Augmented Reality
NEDs	Near-Eye Displays
FOV	Field of View
RGB	Red, Green, Blue
TIR	Total Internal Reflection
SEM	Scanning Electron Microscopy
RCWA	Rigorous Coupled Wave Analysis
DUVL	Deep Ultraviolet Lithography
IC	Integrated Circuit
PECVD	Plasma-Enhanced Chemical Vapor Deposition
EBL	Electron Beam Lithography
PVD	Physical Vapor Deposition
DLP	Digital Light Processing
CMOS	Complementary Metal–Oxide-Semiconductor
NIL	Nanoimprint Lithography
Al	Aluminum
TiN	Titanium Nitride
SiO_2_	Silicon Dioxide
